# Some American Hospitals

**Published:** 1892-02-13

**Authors:** 


### Some American Hospitals.

By Our Special Commissioner.

### VII.—PENNSYLVANIA HOSPITAL.

### Lunatic Department.

This is the oldest hospital in the United States, and the oldest on the North American Continent with one exception, that of the Hospital at Quebec. Like many British hospitals, it was the custom originally to admit insane patients into the wards. After a period of forty years, *i.e.*, from 1756 to 1796, the insane occupied the west wing of the hospital at Pine Street, but in January, 1841, the whole of the lunatics were transferred to the wards of the new Hospital for the Insane in West Philadelphia. The Lunatic Asylum is built on the linear, or Kirkbride plan, which is too well known to need description. The managers take considerable pride in the institution. No paupers are received, and the whole place reflects considerable credit upon all who are connected with it, from the Medical Superintendent (Dr. John B. Chapin) downwards. It appears from his report that the daily average number of patients during the year ending April 22, 1891, was 417, of which 184 were males, and 233 women. Altogether about ten thousand patients have been admitted for treatment since 1841, of whom 4,444 were women and 5,141 were men, from which it would appear that the number of women at the present under treatment is abnormally large. The whole cost of treatment was 27,360 dols., giving an average of 380 dols. (£76) for each patient. There is a Matron (Miss H. P. Saeger) in charge of the asylum, but nothing is said about the training of attendants and nurses, nor of lectures given to the asylum staff.

### The Hospital Proper.

The building of the hospital has a special interest owing to the plan adopted, and to the amount of good work that has been done within it. In a portion of the older hospital the wards have Corinthian pillars on each side, making a passage way of the centre; the beds being placed on the right and left of the pillars on each side of the ward. In one instance the members of the medical staff had taken a great deal of pains to decorate the ward by painting the pillars and placing a few vases of Doulton ware in the central passage, the whole effect being remarkably good. We wish that some well-to-do resident of Philadelphia would visit the hospital and see the ward we refer to, and then we are sure the other wards would soon be made equally attractive. In another portion of the hospital, in lieu of the pillars, side wards have been built between the central passage and the outside walls, so that alcoves have been formed, each of which contains two beds. This arrangement is to be met with in some of the older French hospitals in charge of sisterhoods. It interferes wholly with the free circulation of air, makes it difficult to administer the wards, and is otherwise objectionable. The patients like it because it is possible to get a certain amount of privacy by drawing a curtain in front of each alcove, which is thus made into an ill-ventilated recess, where surgical cases cannot properly be treated at all. There is a smaller children's ward attached to the hospital, which was full, and which reflected the greatest credit upon the nurses in charge, as every one of the little ones was beautifully kept, although some of the cases must give a great deal of trouble from the constant care and attention they require. We also noticed an excellent dressing carriage, of a pattern well worthy of imitation, as it is portable, convenient, and large enough to contain everything which a surgeon or dresser is likely to need in his morning rounds. The hospital contains 225 beds. The average number of patients for the year 1891 has been 164. Managers complain of a want of funds, and regret that they have been compelled to send away cases of great interest because the power of relief was not so readily procurable at the Pennsylvania as it was at other hospitals. They propose to erect new buildings, as they have found the need of wards for isolating gynæcological, diphtheritic, and contagious cases, in addition to rooms for operations, examinations, microscopical, chemical, and surgical work, with improved baths and lavatories. The trustees propose immediately to construct new buildings on four and a-half acres of an unutilised site now in their possession, and which forms a portion of the hospital grounds. No stranger can visit the Pennsylvania Hospital without feeling the deepest interest in its work and welfare. And it is satisfactory to be able to report that everything was in excellent order and beautifully clean. How it has come to pass that the people of Philadelphia have failed to supply the trustees with adequate funds for maintenance, we are at a loss to understand, especially as a wealthy resident who accompanied us put his hand in his pocket more than once during our visit to supply certain things which we suggested as desirable. It sometimes happens that the administrators of an old hospital lack energy to bestir themselves sufficiently to insure that the public are aroused to the necessities of the institutions they control. Whatever the cause may be, it is not only desirable, but necessary, that funds should be given to the trustees of this hospital, so as to enable them to continue and increase the grand work which they and their predecessors have so successfully carried on for more than a century past. More than a third of the total income is derived from the payments of patients in the asylum. The annual expenditure under all heads appears to be about 520,000 dols., and receipts during the year ending March 25th, 1891, amounted to about 560,000 dols.

### Nursing Department.

This department is in charge of Miss Elizabeth Collier, the head nurse, who went to the school rather more than three years ago. She seems to take the liveliest interest in her work, and is most enthusiastic and zealous. We should like some wealthy Philadelphians to visit the nursing department and the home attached to the Pennsylvania Hospital, and see how much it is possible to do to add to the comforts of the nurses by a wise expenditure on the part of a noble-hearted philanthropist like the late Mr. Hana. He gave the nurses a library so well selected that we have thought it desirable to publish a list of the books in the "Nursing Mirror Supplement." Mr. Hana also supplied the nurses' sitting-room with pictures, and furnished it in the most comfortable way. After the wealthy Philadelphians have seen what Mr. Hana has done, we would ask them to visit the Philadelphian Hospital, and also the Hospital of the University of Pennsylvania, with the object of seeing how the nurses' rooms are furnished, and with the determination to supply out of their own pockets anything which may be wanted to make them equally comfortable with those at the Pennsylvania Hospital. There is further need of sleeping accommodation for the nurses of the Pennsylvania Hospital, as some of the rooms at present contain three beds. This may be necessary in cases of probationers, but every such three-bedded room should contain three screens, so as to secure necessary privacy. As the sleeping-rooms and training-rooms at the Pennsylvania Hospital contained no screens, Mr. Drexel, jun., who accompanied us at the time of our visit, generously consented to provide them at once, and to pay for them. Mr. Drexel also undertook to supply the nurses' sitting-room with three papers, The Hospital, the *Trained Nurse*, and the *Nightingale*. Of course, the expense of an act of kindness like this is small, but it would be well if some lady or gentleman would interest themselves sufficiently in hospital nurses, near where they reside, and would take the trouble to supply the nurses' library of each such institution with periodical literature in the same way.

